# Supplementary material for: Development of a computer-aided design software for the quantitative evaluation of aesthetic damage
Source: PLoS One. 2019 Dec 18;14(12):e0226322. doi: 10.1371/journal.pone.0226322 (PMC6919621; doi:10.1371/journal.pone.0226322)
Supplement: S1 Code site — 1. (ZIP) [file pone.0226322.s001.zip › [EESC_jr][Projeto]Relatorio_07_21/reports/templates_zip/gentelella-master/vendors/select2/docs/options-old.html]

---
layout: default
title: Options - Select2
slug: options
---

# Options

{% include options-old/core-options.html %}
{% include options-old/dropdown.html %}
{% include options-old/events.html %}
{% include options-old/adapters.html %}
{% include options-old/setting-default-options.html %}
{% include options-old/backwards-compatibility.html %}

{% include nav/options-old.html %}
